# The Role of Hydrogen Sulfide in Renal System

**DOI:** 10.3389/fphar.2016.00385

**Published:** 2016-10-18

**Authors:** Xu Cao, Jin-Song Bian

**Affiliations:** Department of Pharmacology, Yong Loo Lin School of Medicine, National University of SingaporeSingapore, Singapore

**Keywords:** hydrogen sulfide, H_2_S, renal physiology, acute kidney injury, chronic kidney disease, diabetic nephropathy

## Abstract

Hydrogen sulfide has gained recognition as the third gaseous signaling molecule after nitric oxide and carbon monoxide. This review surveys the emerging role of H_2_S in mammalian renal system, with emphasis on both renal physiology and diseases. H_2_S is produced redundantly by four pathways in kidney, indicating the abundance of this gaseous molecule in the organ. In physiological conditions, H_2_S was found to regulate the excretory function of the kidney possibly by the inhibitory effect on sodium transporters on renal tubular cells. Likewise, it also influences the release of renin from juxtaglomerular cells and thereby modulates blood pressure. A possible role of H_2_S as an oxygen sensor has also been discussed, especially at renal medulla. Alternation of H_2_S level has been implicated in various pathological conditions such as renal ischemia/reperfusion, obstructive nephropathy, diabetic nephropathy, and hypertensive nephropathy. Moreover, H_2_S donors exhibit broad beneficial effects in renal diseases although a few conflicts need to be resolved. Further research reveals that multiple mechanisms are underlying the protective effects of H_2_S, including anti-inflammation, anti-oxidation, and anti-apoptosis. In the review, several research directions are also proposed including the role of mitochondrial H_2_S in renal diseases, H_2_S delivery to kidney by targeting D-amino acid oxidase/3-mercaptopyruvate sulfurtransferase (DAO/3-MST) pathway, effect of drug-like H_2_S donors in kidney diseases and understanding the molecular mechanism of H_2_S. The completion of the studies in these directions will not only improves our understanding of renal H_2_S functions but may also be critical to translate H_2_S to be a new therapy for renal diseases.

## Introduction

Hydrogen sulfide has been regarded as a toxic gas for 100s of years (Smith and Gosselin, 1979). It can directly inhibit the activity of several essential enzymes in human namely cytochrome c oxidase (Reiffenstein et al., 1992), carbonic anhydrase (Nicholson et al., 1998), monoamine oxidase (Warenycia et al., 1989), and Na^+^/K^+^ ATPase (Reiffenstein et al., 1992), thereby causing toxicity. However, the image of H_2_S has been largely expanded since the revelation of H_2_S as an endogenous neuronal modulator by Kimura’s group in Abe and Kimura (1996). Thereafter, the physiological significance of H_2_S has been extensively studied especially in central nervous system (Zhang and Bian, 2014) and cardiovascular system (Liu et al., 2012). Emerging evidence has suggested that H_2_S also actively regulates renal function and is implicated in numerous kidney diseases in recent years. Here in this review, recent studies regarded the role of H_2_S in both kidney physiology and diseases will be discussed.

## Physical and Chemical Properties of H_2_S

Hydrogen sulfide exists as a colorless gas with a strong rotten egg smell at room temperature and ambient pressure. The human nose can detect a concentration of 400-fold lower than its toxic level (Wang, 2002), whereas, long term exposure can cause desensitization of olfactory nerves to H_2_S (Li et al., 2009). Distinct from the other gaseous transmitters like NO and CO, H_2_S is a weak acid and hence able to readily dissolve in water. Based on its PKa, it is estimated that there will be 14% H_2_S gas, 86% HS^-^ and a trace of S^2-^ in physiological condition (pH 7.4, 37°C; Li et al., 2009). Moreover, H_2_S gas is highly lipophilic which allows it freely to penetrate into the cell membrane of all types and become biologically active (Mathai et al., 2009).

## H_2_S Generation in the Kidney

Three traditional H_2_S synthesizing pathways have been identified in mammalians including CSE (EC 4.4.1.1), CBS (EC 4.2.1.22), and 3-MST (EC 2.8.1.2) coupled with CAT (EC 2.6.1.3) pathways. The mechanisms underlying these traditional pathways can be found in our previous review in detail (Liu et al., 2012). In short, CSE firstly dimerizes two L-cysteine to L-cystine followed by transforming it into pyruvate, NH_3_ and thiocysteine. The resulted thiocystein is then used as a substrate by CSE to react with other thiols to generate H_2_S (Stipanuk and King, 1982). CBS catalyzes the reaction between L-cysteine and homocystenin into cystathinine and H_2_S (Szabo, 2007). However, 3-MST is unable to directly use L-cysteine as a substrate as its counterpart does. L-cysteine has to be transformed into 3-MP by CAT which is then catalyzed by 3-MST into pyruvate and H_2_S (Shibuya et al., 2009). It is worth mentioning that both CSE and CBS require pyridoxal 5′-phosphate as a cofactor to synthesize H_2_S, while 3-MST is dependent on zinc (Li et al., 2009). In addition, CSE and CBS mainly locate in cytosol yet they can translocate into mitochondria in some oxidative conditions (Fu et al., 2012), whereas 3-MST resides and generates H_2_S in mitochondria (Kimura, 2011).

A fourth H_2_S generation pathway namely DAO/3-MST pathway was discovered recently by Kimura’s group (Shibuya et al., 2013). In the study, they showed that kidney lysate can produce 60 times more H_2_S when using D-cysteine as substrate comparing with L-cysteine. Further, the underlying mechanism was studied. Specifically, D-cysteine is transformed into 3-MP by peroxisome located DAO. Due to metabolite exchanges between peroxisome and mitochondria, 3-MP is imported into mitochondria and catalyzed into H_2_S by 3-MST. Since DAO is only located in brain and kidney, this H_2_S generating pathway is believed to exclusively exist in brain and kidney.

**FIGURE 1:**
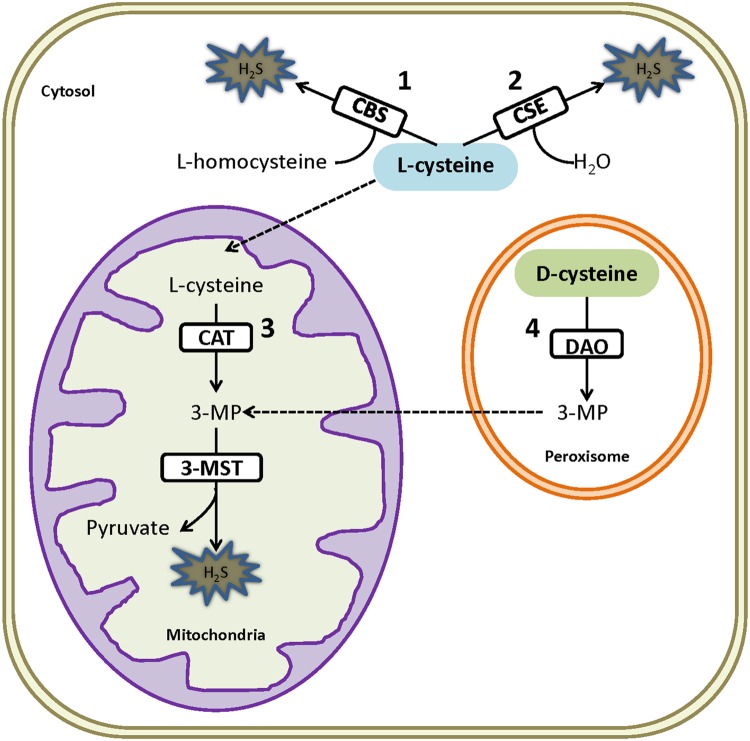
**Endogenous synthesis of H_2_S by four pathways in renal system.** (1) CBS mediated H_2_S synthesis by using L-cysteine as the substrate in cytosol; (2) CSE mediated H_2_S synthesis with L-cysteine as the substrate in cytosol; (3) CAT transforms L-cysteine into 3MP which is further catalyzed by 3-MST into H_2_S in mitochondria; (4) Peroxisome resident DAO catalyzes D-cysteine into 3MP which is then exchanged into mitochondria and utilized by 3-MST for the production of H_2_S in mitochondria. CBS, cystathionine β-synthase; CSE, cystathionine γ-lyase; 3-MST, 3-mercaptopyruvate sulfurtransferase; CAT, cysteine aminotransferase; DAO, D-amino acid oxidase; 3MP, 3-mercaptopyruvate.

Hydrogen sulfide generation is abundant in kidney given the presence of all the above mentioned pathways in this organ (**Figure 1**). Currently, it is believed that CSE and CBS are the dominated enzymes for H_2_S generation in kidney. The presence of these two enzymes was firstly demonstrated in 1980s by using their inhibitors (Stipanuk and Beck, 1982). Later on, House et al. (1997) suggested that both enzymes were mainly located on renal proximal tubules within the renal cortex by comparison with marker enzymes of known location. This finding was supported by several other studies using different methods (Ishii et al., 2004; Li et al., 2006; Tripatara et al., 2009). However, inconsistent results have been reported regarding the existence of these two enzymes in glomerulus which needs to be resolved (Aminzadeh and Vaziri, 2012; Bos et al., 2013; Yamamoto et al., 2013). In addition, definitive evidence has suggested the presence of 3-MST in kidney (Aminzadeh and Vaziri, 2012; Shibuya et al., 2013; Kimura, 2014; Pan et al., 2015), however, the significance of 3-MST mediated H_2_S generating pathway has not been well-acknowledged in both kidney physiology and diseases due to limited reports. Nevertheless, the revelation of the unique DAO/3-MST pathway in kidney and brain may imply a significant role of 3-MST mediated H_2_S generation in these organs. This will be an interesting area open to explore in the next years.

## Effect of H_2_S On Renal Physiology

### H_2_S Effect on Renal Excretory Function

**FIGURE 2:**
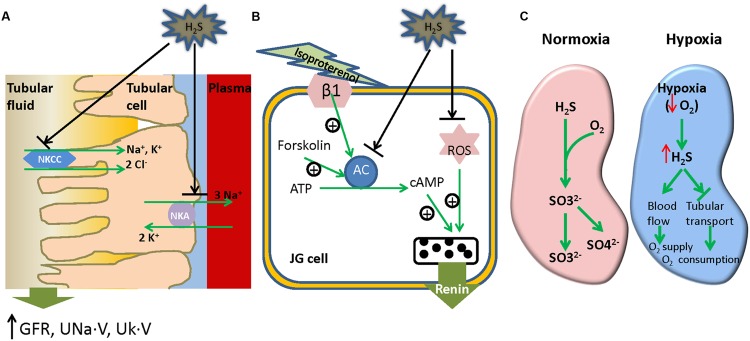
**Effects of H_2_S on renal regulation. (A)** H_2_S inhibits the activity of tubular NKCC and NKA, thereby enhancing renal excretory function such as GFR, UNa.V and Uk.V. **(B)** H_2_S suppresses renin release by inhibition of AC activity and ROS production in GC cells. **(C)** H_2_S as an O_2_ sensor in the kidney. In normoxic condition, H_2_S is metabolized into sulfates in the presence of O_2_; in hypoxic condition, the shortage of O_2_ leads to the accumulation of H_2_S which helps to restore O_2_ level by enhancing blood flow and suppressing tubular transport activity in kidney. NKCC, Na^+^/K^+^/2Cl^-^ cotransporter; NKA, Na^+^/K^+^ ATPase; GFR, glomerular filtration rate; UNa⋅V, urinary sodium; Uk⋅V, urinary potassium; β1, β1-adrenoceptor; ROS, reactive oxygen species; ATP, adenosine triphosphate; cAMP, cyclic adenosine monophosphate; JG cell, juxtaglomerular cell.

The necessity of H_2_S producing enzymes have long been recognized in the kidney due to their critical effect on homocysteine metabolism (Stipanuk, 2004), however, the effect of H_2_S itself on renal function was not studied until recently. Intra-renal infusion of H_2_S donor NaHS is able to increase GFR, UNa⋅V and potassium (Uk⋅V) excretion (Xia et al., 2009; Ge et al., 2014). Moreover, the effect is closely mimicked by the infusion of L-cysteine, an H_2_S generating substrate (Xia et al., 2009). In addition, inhibition of endogenous H_2_S production by AOAA (CBS inhibitor) plus PAG (CSE inhibitor) leads to the decrease of GFR, UNa⋅V and Uk⋅V, suggesting that H_2_S regulates renal function in physiological conditions. However, either AOAA or PAG alone fails to produce any effect on renal function implicating a compensatory effect between CBS and CSE on renal regulation which has been confirmed by another study (Roy et al., 2012). Hypothesis concerning the effects of H_2_S on sodium transporters was formed and tested thereafter. The results showed that H_2_S significantly inhibited the activity of NKCC and NKA which may account for its effect on renal function (**Figure 2A**). Recently, the mechanism of the inhibitory effect of H_2_S on NKA was studied by Zhu’s group (Ge et al., 2014). In their study, they showed that NaHS promoted NKA endocytosis by directly activating epidermal growth factor receptor (EGFR) in renal tubular epithelia cells. Moreover, EGFR cys797 mutation fully abolished the effect of H_2_S suggesting a direct interaction between H_2_S and this cysteine residue. Taken together, both endogenous and exogenous H_2_S are able to increase GFR, UNa⋅V, and Uk⋅V excretion probably through the inhibitory effect on sodium transporters like NKCC and NKA.

Hydrogen sulfide increases Cl^-^/HCO_3_^-^ exchanger activity in various tissues like aortic tissues (Liu and Bian, 2010) and vascular smooth muscle cells (Lee et al., 2007). However, the effect of H_2_S on the exchanger in kidney is still unknown. It is likely that H_2_S can also enhance the activity of Cl^-^/HCO_3_^-^ exchanger in renal system. Considering the critical role of Cl^-^/HCO_3_^-^ exchanger in regulating the excretion of ions and homeostatic maintenance of physiological pH, it will be of great value to reveal the effect of H_2_S on Cl^-^/HCO_3_^-^ exchanger activity and the subsequent consequence in kidney.

### H_2_S Effect on Renin Release

The RAS is a renovascular hormone system involved in the regulation of plasma sodium concentration and blood pressure (BP). Renin release from JG cells determines RAS activity and the process is known to be regulated by intracellular cAMP (Peters et al., 1993; Gambaryan et al., 1998; Schweda et al., 2007). Moreover, H_2_S was reported to downregulate cAMP level in several cell types (Lim et al., 2008; Yong et al., 2008), implying that H_2_S may modulate renin release. This was demonstrated by our group in Lu et al. (2010). It was found that NaHS inhibited the upregulation of renin mRNA and protein level in a model of renovascular hypertension accompanying with a reduction of intracellular cAMP level. This is supported by another study (Lu et al., 2012) showing that H_2_S regulates renin degranulation in As4.1 and rat renin-rich kidney cells stimulated by isoproterenol, forskolin or 3-isobutyl-1-methylxanthine. Further study demonstrated that H_2_S significantly suppressed the stimulated AC activity in these cells. Overexpression of CSE also attenuates isoproterenol-induced renin release suggesting that endogenous H_2_S may also involve in the process. However, the mechanism underlying the inhibitory effect of H_2_S on AC remains to be determined, such as the identification of AC isoform(s) accounting for H_2_S effect and the molecular interaction between H_2_S and the AC isoform(s). Besides AC, ROS was recently reported to be a target of H_2_S for its effect on renin reduction in DN suggesting participation of multiple mechanisms in the process (Xue et al., 2013). The effect of H_2_S on renin activity in normal rats has also been investigated (Lu et al., 2010). The results showed that neither NaHS administration nor inhibition of endogenous H_2_S influenced renin activity implying that H_2_S may only modulate renal activity when RAS is overactivated. The effect of H_2_S on renin activity has been illustrated in **Figure 2B**.

### H_2_S as an O_2_ Sensor in the Kidney

Essentially all H_2_S generation is independent of O_2_; however, the metabolism of H_2_S is a process highly relying on O_2_ (Olson, 2015). Accumulating evidence suggests that H_2_S is an O_2_ sensor in kidney, especially in medulla (**Figure 2C**). The availability of O_2_ in renal medulla is lower compared with that in renal cortex resulting in a higher abundance of H_2_S in this region (Koning et al., 2015). Provided mitochondria can use H_2_S as an electron donor for ATP production (Fu et al., 2012; Teng et al., 2013), it will be interesting to hypothesize that H_2_S might be a direct source of energy in renal medulla. During hypoxia, O_2_ reduction leads to further accumulation of H_2_S which help to recover O_2_ supply by increasing medullary blood flow and inhibition of tubular transport (Beltowski, 2010). In addition, CBS and CSE can translocate into mitochondria and stimulate H_2_S production under hypoxic circumstances (Fu et al., 2012; Teng et al., 2013). The mitochondria derived H_2_S may directly participate in ATP production. Currently, emerging physiological evidence for H_2_S mediated O_2_ sensing has also been suggested in various O_2_ sensing tissues including cardiovascular system (Olson et al., 2006; Olson and Whitfield, 2010), respiratory system (Hu et al., 2008), gastrointestinal tract (Dombkowski et al., 2011) et al. However, the downstream effectors of H_2_S mediated O_2_ sensing remains to be determined.

## H_2_S in Acute Kidney Injury

Acute kidney injury (formerly known as acute renal failure) is defined as a syndrome characterized by rapid loss of the kidney’s excretory function. It is the clinical manifestation of several disorders that affect the kidney acutely (Bellomo et al., 2012). Here, H_2_S effects in three types of acute kidney injury will be discussed namely renal IRI, obstructive nephropathy, and cisplatin nephrotoxicity.

### H_2_S in Renal Ischemia/Reperfusion Injury

Renal IRI is a major cause of acute kidney injury. The pathophysiological mechanism underlying renal IRI is very complex containing ATP depletion, calcium overload, ROS generation, apoptotic and inflammatory responses et al (Eltzschig and Eckle, 2011). The engagement of endogenous H_2_S in renal IRI has been thoroughly demonstrated in various studies. Specifically, both mRNA and protein levels of CSE and CBS are apparently reduced upon IRI along with the reduction of H_2_S level in kidney and plasma (Xu et al., 2009; Han et al., 2015), although mechanisms underlying IRI caused CSE and CBS reduction are still not revealed. In addition, inhibition of either CSE or CBS by their pharmacological inhibitors severely aggravates renal damage (Tripatara et al., 2008; Han et al., 2015) indicating that the ischemic renal injury might, at least in part, results from the impaired endogenous production of H_2_S. The implication is supported by a recent finding that CSE-deficiency associates with increased renal damage and mortality after renal IRI which might be due to the enhanced production of ROS (Bos et al., 2013). Subsequently, the effect of exogenous H_2_S was extensively studied in various renal IRI scenarios (Tripatara et al., 2008, 2009; Bos et al., 2009, 2013; Xu et al., 2009; Simon et al., 2011; Hunter et al., 2012; Zhu et al., 2012; Azizi et al., 2015; Han et al., 2015; Ahmad et al., 2016) which have been summarized in **Table 1**. In most studies, an H_2_S donor, NaHS, was employed and exerted protective effects likely through anti-inflammatory, anti-apoptotic, and anti-oxidative responses. Comparing with new generation synthetic H_2_S donors like GYY4137, NaHS is less physiologically accurate H2S producer (Li et al., 2008; Yu et al., 2010). Recently, GYY4137 was shown to attenuate heart damage by inhibiting activation of NF-κB and MAPK signaling in a rat model of myocardial IRI (Meng et al., 2015a,b). Thus, studies are warranted to study whether slow H_2_S donors like GYY4137 can protect kidney form IRI. Besides, it is worth noting that AP39, a mitochondrially targeted donor of H_2_S, was recently found to inhibit intracellular ROS formation caused by glucose oxidase and protect kidney from IRI caused damage in rats (Ahmad et al., 2016). The study implies the importance of mitochondria H_2_S in the pathology of renal IRI which needs to be determined in the future.

**Table 1:** Comparison of the renal protective effect of H_2_S against ischemic/reperfusion injury.

Treatment	I/R protocol	Species/tissue	Effects of H_2_S	Mechanism	Reference
NaHS (100 μmol/kg, i.p., 30 min prior to ischemia)	I (30 min)/R (24 h)	C57BL/6 mice	Improved renal function	–	Tripatara et al., 2008
NaHS (1 mg/kg, i.p., 15 min prior to ischemia)	I (30 min)/R (24 h)	C57BL/6 mice	Improved renal function; reduced renal injury, and mortality	Anti-oxidation	Bos et al., 2009
NaHS (100 μg/kg, i.p., 15 min prior to ischemia)	I (45 min)/R (6 h)	Sprague-Dawley rat	Improved renal function; reduced renal injury	Anti-oxidation; anti-apoptosis	Tripatara et al., 2009
NaHS (500 μg/kg, i.p., first dose at 2 days after ischemia; then daily)	I (30 min)/R (8 days)	C57BL/6 mice	Accelerated kidney recovery and tubular cell regeneration	Anti-oxidation	Xu et al., 2009
NaHS (100 μmol/kg, topically onto the kidneys 15 min before ischemia and 5 min before reperfusion)	I (45 min)/R (6 h)	Wistar rat	Improved renal function; reduced renal injury	Anti-apoptosis; anti-MAPK; anti-NF-kB	Simon et al., 2011
H2S (100 ppm, 30 prior to ischemia)	I (30 min)/R (24 h)	C57BL/6 mice	Improved renal function; reduced renal injury	Induction of hypometabolism	Hunter et al., 2012
Na_2_S (initial bolus 0.2 mg/kg followed by continuous i.v. 2 mg/kg/h during the 2 h before aortic occlusion, 0.5 mg/kg/h during the 90 min of aortic occlusion, and 1 mg/kg/h during the 8-h reperfusion period)	I (2 h)/R (8 h)	Local pig	Improved renal function; reduced renal injury	Anti-apoptosis; anti-oxidation; anti-NF-kB	Zhu et al., 2012
NaHS (150 μmol/kg, i.p., 30 min prior to ischemia)	I (1 h)/R (2 h); warm ischemia	Lewis rat	Improved renal function; reduced renal injury	Anti-inflammation	Bos et al., 2013
Na_2_S (i.v.; a bolus of 100 μg/kg was given 10 min before reperfusion, followed by an infusion of 1 mg/kg given continuously for 30 min after reperfusion)	I (1 h)/R (7 days)	Large white pig	Improved renal function; reduced renal injury	Anti-inflammation	Azizi et al., 2015
NaHS (75 μmol/kg; i.p.; 10 min prior to ischemia and immediately before reperfusion)	I (55 min)/R (24 h)	Wistar rat	Improved renal function; reduced renal injury	Anti-apoptosis; anti-oxidation	Han et al., 2015
AP39 (0.3 mg/kg; i.p.; 5 min before reperfusion)	I (30 min)/R (24 h)	Sprague-Dawley rat	Improved renal function; reduced renal injury	Anti-apoptosis; anti-oxidation	Ahmad et al., 2016

*i.p., intraperitoneal; i.v., intravenous; I/R, ischemia/reperfusion; MAPK, mitogen-activated protein kinase.*

### H_2_S in Obstructive Nephropathy

Obstructive nephropathy is a type of renal injury caused by obstruction of the genitourinary tract. Renal fibrosis after ureteral obstruction is implicated in the development of obstructive nephropathy (Boor et al., 2010). Hu’s group showed that ureteral obstruction impaired endogenous production of H_2_S by reducing the expression level of CBS (Song et al., 2014). Renal fibrosis is attenuated when exogenous H_2_S is administered suggesting an inhibitory effect of H_2_S on renal fibrosis (Song et al., 2014). In cultured kidney fibroblast, NaHS is able to inhibit cell proliferation and block the differentiation into myofibroblasts by suppressing transforming growth-β1-Smad and MAPK signaling pathways (Song et al., 2014). Furthermore, administration of NaHS also prevents the disruption of renal function caused by ureteral obstruction (Jiang et al., 2014; Song et al., 2014; Dursun et al., 2015). A recent study from Sener’s group (Lin et al., 2016) showed that H_2_S slow releasing donor GYY4137 mitigated cortical loss, inflammatory damage and tubulointerstitial fibrosis in a rat model of obstructive nephropathy. Taken together, these results suggest a potential use of H_2_S donor as a rescue in obstructive nephropathy.

### H_2_S in Cisplatin Nephrotoxicity

Cisplatin is a major therapeutic drug for solid tumors, but causes severe nephrotoxicity. Over 30% of patients receiving high dose cisplatin develop renal dysfunction (Pabla and Dong, 2008). However, effective treatment of cisplatin induced renal failure is still lacking. Extensive research revealed that oxidative stress and inflammatory response are the major driving forces for cisplatin induced renal toxicity (Pabla and Dong, 2008; Peres and da Cunha, 2013). Given the well-known inhibitory effects of H_2_S on oxidative stress and inflammation (Łowicka and Bełtowski, 2006), it is reasonable to hypothesize that H_2_S is protective against cisplatin nephrotoxicity. However, the role of H_2_S is rather controversial due to conflicting data in this field. Coimbra and others (Della Coletta Francescato et al., 2011) firstly showed that cisplatin upregulated the expression level of CSE after 3 days upon cisplatin treatment in a rat model. When PAG was administered with cisplatin, it was found that PAG abolished the upregulation of CSE and rescued cisplatin caused renal damage by suppressing inflammation and apoptosis (Della Coletta Francescato et al., 2011). In contrast to this study, administration of NaHS ameliorates the kidney dysfunction and damage in cisplatin treated rat (Ahangarpour et al., 2014). Liu et al. (2016) recently showed that both CSE and CBS levels were severely decreased upon cisplatin treatment in mouse after 3 days. However, pretreatment with H_2_S slow releasing donor GYY4137 aggravates cisplatin induced renal damage by increasing inflammatory response (Liu et al., 2016). However, several defects on the use of GYY4137 need to be pointed out in the study. As they used a rather low dose of GYY4137 (21 mg/kg), it is possible that H_2_S might be not generated sufficiently (Li et al., 2008; Yu et al., 2010; Meng et al., 2015a,b; Lin et al., 2016). In addition, to rule out the possibility that the chemical backbone of GYY4137 molecule has aggravated cisplatin nephrotoxicity, ZYJ1122 (Lee et al., 2011), a structure analog of GYY4127, should be included for the study. Despite the promising protective effect of H_2_S, however, it is still far from the conclusion that H_2_S is protective in cisplatin nephrotoxicity. In future, more studies are still needed to further investigate (1) the change of CSE and CBS expression level in a time course dependant manner; (2) the effect of endogenous H_2_S by using genetic mouse rather than non-specific CBS/CSE inhibitors; (3) the effect of exogenous H_2_S by using different H_2_S (NaHS, GYY4137, AP39) donors in parallel.

## H_2_S in Chronic Kidney Disease

Chronic kidney disease is a general term for heterogeneous disorders affecting kidney structure and function. In western countries, it is generally associated with old age, diabetes, hypertension, obesity, and cardiovascular disease (Levey and Coresh, 2012). Diabetic nephropathy and hypertensive nephropathy are considered as presumed pathological entities. The role of H_2_S in these two types of CKD will be reviewed below.

### H_2_S in Diabetic Nephropathy

**FIGURE 3:**
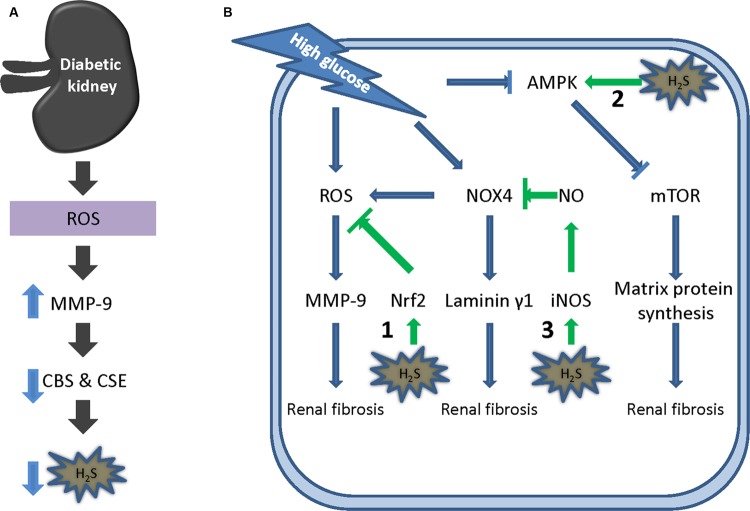
**Hydrogen sulfide in DN. (A)** ROS mediated MMP-9 up-regulation reduces the level of CBS and CSE in DN. **(B)** Mechanisms underlying the protective effect of H_2_S in DN. (1) H_2_S inhibits ROS formation by activating Nrf2 pathway; (2) H_2_S activates AMPK, thereby suppressing PI3K/Akt/mTORC1 signaling and subsequent protein synthesis; (3) H_2_S stimulates NO formation by induction of iNOS expression which inhibits NOX4 level and ROS production. ROS, reactive oxygen species; MMP-9, matrix metalloproteinase-9; AMPK, AMP-activated protein kinase; NO, nitric oxide; iNOS, inducible nitric oxide synthase; mTOR, mechanistic target of rapamycin.

Diabetic nephropathy is the number one leading cause of CKD in western countries. Morphologically, DN is characterized by hypertrophy induced kidney growth and excessive accumulation of extracellular matrix proteins, eventually proceeding to fibrosis of glomerular and tubulointerstitial compartments (Cooper, 1998; Dronavalli et al., 2008). Current evidence suggests an active role of H_2_S in the pathogenesis of DN. Plasma H_2_S level in DN patients is significantly lower than that in non-DN patients undergoing chronic hemodialysis (Li et al., 2014). High urinary sulfate concentration, a reflection of high plasma H_2_S level, is associated with reduced risk of renal disease progression in type 2 diabetes (van den Born et al., 2016) and slower decline in Cr^51^-EDTA-assessed GFR in DN patients (Andresdottir et al., 2013). Recent data suggested that the renal expression of H_2_S producing enzyme CBS and CSE is down-regulated in pancreatic CaMTg diabetic mice (Yamamoto et al., 2013), C57BL/KsJ lepr^-/-^ db/db mice (Lee et al., 2012), STZ-diabetic rats (Yuan et al., 2011), and Akita diabetic mice (Kundu et al., 2013). Inhibition of CSE with PAG mimics high glucose-induced glomerular podocyte injury (Liu et al., 2015), implying a contributive role of endogenous H_2_S in DN. The mechanisms underlying CBS and CSE reduction have been also studied. Kundu et al. (2013) showed that MMP-9 was upregulated in Akita diabetic mice along with the reduction of CBS and CSE. When MMP-9 is knocked out, the expression of the two H_2_S producing enzyme namely CSE and CBS shows a trend toward baseline despite hyperglycemia (Kundu et al., 2013) suggesting that MMP-9 regulates CBS and CSE expression in DN (**Figure 3A**).

Meanwhile, exogenous H_2_S has been proved to be effective in *in vitro* and *in vivo* DN models. High glucose caused cell proliferation and collagen formation are attenuated by NaHS in cultured messangial cells (Yuan et al., 2011) and tubular cells (Safar and Abdelsalam, 2015). Moreover, NaHS alone (Yuan et al., 2011; Ahmad et al., 2012; Lee et al., 2012; Kundu et al., 2013; Xue et al., 2013; Zhou et al., 2014; Liu et al., 2015; Safar and Abdelsalam, 2015; Qian et al., 2016) or with losartan (Kaur et al., 2015) ameliorates renal dysfunction and fibrosis formation in various DN related animal models. At least three mechanisms (**Figure 3B**) are implicated in H_2_S mediated protective effect in DN: (1) Inhibition of ROS formation by activating Nrf2 pathway. Hyperglycemia induces intracellular ROS which upregulates the expression of MMP-9 (Kundu et al., 2013). MMP-9 plays a major role in diabetic renovascular remodeling. In a STZ induced diabetic rat model, H_2_S was found to reduce high glucose induced oxidative stress by activating the Nrf2 antioxidant pathway and thereby the level of MMP-9 (Zhou et al., 2014); (2) AMPK activation. Matrix protein deposition requires stimulation of protein synthesis. In DN, PI3K/Akt/mTORC1 signaling is activated due to the suppression of AMPK activity (Yuzawa, 2012). The inactivation of AMPK was found to be partially ascribed to the reduction of H_2_S level (Lee et al., 2012). NaHS dose- and time- dependently activates the phosphorylation of AMPK and inhibits the stimulation of mTOR (Lee et al., 2012). As a result, mTOR mediated protein synthesis is inhibited which partially accounts for the protective effect of H_2_S in DN. (3) Stimulation of NO formation. Hyperglycemia upregulates NOX4 expression resulting in the generation of intracellular ROS and laminin γ1, both of which contribute to renovascular remodeling (Gorin et al., 2015). Expression of NOX4 can be attenuated by H_2_S (Kamat et al., 2015). L-NAME, a NOS inhibitor, abolished the effect of H_2_S (Feliers et al., 2016). Further study showed that H_2_S upregulates the protein expression of iNOS but not that of eNOS (Feliers et al., 2016). Moreover, iNOS siRNA can block the effect of H_2_S (Feliers et al., 2016) suggesting a role for NO in H_2_S mediated protective effect in DN. Interestingly, NO can also stimulate the activity of CSE and H_2_S production (Nagpure and Bian, 2016) suggesting a cross-talk interaction between these two pathways.

### H_2_S in Hypertensive Nephropathy

Hypertensive nephropathy, a result of chronic hypertension, is the second leading cause of CKD worldwide (Hart and Bakris, 2010). Long time of high BP load results in damages of kidney, especially in glomerulus. When kidney cannot function properly, it fails to regulate BP. In turn, BP will rise up which further aggravates renal damage (Bidani and Griffin, 2004). Extensive evidence suggests a role of H_2_S in BP control. Genetic deletion of CSE causes hypertension and diminished endothelium dependent vasorelaxation (Yang et al., 2008). Similar with this, inhibition of both CBS and CSE also increases BP in rat (Roy et al., 2012). These data implies that H_2_S is a physiologic regulator of BP. Further studies suggest that H_2_S functions as both an EDHF (Mustafa et al., 2011; Edwards et al., 2012; Tang et al., 2013) and an EDRF (Wang, 2009). The BP lowering effect of exogenous H_2_S has been subsequently determined. Our group first showed that H_2_S donor, NaHS, attenuated BP by inhibiting plasma renin activity in a 2K1C rat model (Lu et al., 2010). Thereafter, NaHS was demonstrated to reduce BP in spontaneous hypertensive rats (Ahmad et al., 2014), angiotensin II treated mice (Al-Magableh et al., 2015), and sFlt transgenic mice (Holwerda et al., 2014). Additionally, renal protective effects of H_2_S have also been reported in hypertensive nephropathy. sFlt overexpression in mouse results in hypertension with proteinuria and glomerular endothelosis, all of which are apparently attenuated by administration of NaHS (Holwerda et al., 2014). Further study suggests an involvement of VEGF in this H_2_S mediated effect (Holwerda et al., 2014). Both H_2_S and tempol were found to alleviate renal dysfunction in a spontaneous hypertensive rat model by suppressing ROS formation (Ahmad et al., 2014). Recently, Jin’s group reported that either NaHS or its metabolite sodium thiosulfate attenuated angiotensin II induced proteinuria, renal dysfunction, and structural deterioration in rat (Al-Magableh et al., 2015). Further studies suggested that the renal effects of H_2_S were partially mediated by suppression of epithelial sodium channel (Zhang et al., 2013; Wang et al., 2015). Taken together, H_2_S might be an ideal candidate for the treatment of hypertensive nephropathy.

## Future Directions

### Role of Mitochondrial H_2_S Pathway in Kidney Diseases

In contrast to the extensive studies on CBS and CSE in various kidney diseases, the role of 3-MST is largely neglected although definitive evidence has demonstrated its abundance in kidney (Aminzadeh and Vaziri, 2012; Shibuya et al., 2013; Kimura, 2014). 3-MST resides in mitochondria and is the major producer of mitochondria derived H_2_S. 3-MST silencing was reported to reduce bioenergetic parameters and H_2_S can serve as an electron donor in mammalian cells (Modis et al., 2013), indicating a possible physiological role of 3-MST/H_2_S pathway in maintaining mitochondrial electron transport and cellular bioenergitics. Renal ischemia is the most common cause of acute kidney injury. Hypoxia leads to the inhibition of mitochondrial respiratory chain by deprivation of O_2_. What will happen if H_2_S is supplemented into the mitochondria when hypoxia occurs? Szabo’s group (Ahmad et al., 2016) showed that mitochondrially targeted H_2_S donor AP39 ameliorated renal damage in an ischemia/reperfusion rat model, suggesting possible involvement of 3-MST/H_2_S pathway in the pathogenesis although the change of 3-MST level and activity upon hypoxia was not determined. Additionally, the importance of 3-MST/H_2_S pathway is also suggested by the fact that ROS, the common cause of renal diseases of all types, induces the translocation of CBS and CSE into mitochondria (Fu et al., 2012). Thus, the revelation of the involvement of 3-MST in kidney diseases is of great value.

### Targeting DAO/3-MST Pathway for H_2_S Delivery to Kidney

Safety is always a concern when administrating H_2_S systemically due to its well-known toxicity (Guidotti, 2010; Hirose, 2010) which might hamper its development as therapeutics. This safety issue might be diminished by specific delivery of H_2_S to the targeted organs. Unfortunately, such organ specific H_2_S donor is not reported. The discovery of DAO/3-MST pathway might provide a clue about how to deliver H_2_S specifically into the kidney. The pathway uniquely utilizes D-cysteine rather than L-cystein to produce H_2_S and exclusively exists in cerebellum and kidney (Shibuya et al., 2013). When D-cysteine is given, it attenuates IRI in kidney with higher potency than L-cysteine (Shibuya et al., 2013). As H_2_S is broadly renal protective as we have reviewed above, D-cysteine might also ameliorate other renal diseases. Direct administration of D-cysteine induces the generation of H_2_S in both cerebellum and kidney (Shibuya et al., 2013). Nevertheless, it is possible that structural modification of D-cysteine can generate a novel moiety providing D-cysteine to kidney but not cerebellum due to the impermeability of blood–brain barrier. This hypothesis warrants further investigation.

### Test of Drug-Like H_2_S Donors in Kidney Disease

To date, most studies of H_2_S effect in kidney have been largely restricted to the use of NaHS as an H_2_S donor despite of rare exceptions with GYY4137 or AP39. However, NaHS releases H_2_S at an uncontrolled manner (Li et al., 2008) and is unlikely to be a therapeutic agent (Szabo, 2007). Numerous drug-like H_2_S donors have been developed and some of them are under the investigation in clinical trials. For instance, an orally active H_2_S donor SG-1002 is proven to be safe in humans and underwent Phase II study for heart failure (ClinicalTrials.gov identifier: NCT01989208); Antibe Therapeutics are conducting several preclinical or clinical studies with their various H_2_S donors (ATB-346 for osteoarthritis; ATB-352 for Acute pain; ATB-350 for Thrombosis). The information on these H_2_S releasing donors can be found in Wallace and Wang (2015). Testing of these drug-like H_2_S donors will not only consolidate the protective effect of H_2_S, but also shed light on the translation of H_2_S as a therapeutic agent for renal diseases.

### Understanding the Molecular Mechanism of H_2_S

Last, but not least, one should bear in mind that the molecular mechanisms underlying H_2_S effect is still not well-understood. It seems that H_2_S may partially exert its effect as a reducing agent to eliminate ROS (Bruce King, 2013), while its effects on gene expression may be related to specific molecular targets like NF-κB and the ERK pathways (Oh et al., 2006). However, the molecular details are still unclear. Thus, the in-depth portrayal about the interaction between H_2_S and its target protein will be interesting.

## Conclusion

After recognition as the third gaseous mediator after NO and CO, the biological actions of H_2_S are still expanding. In kidney, it is actively participating in the renal regulation in physiological condition. Due to the regulatory role of kidney in the body, it is possible that H_2_S has far-reaching actions by modulating renal function which needs to be uncovered. Because of the significant role of H_2_S in renal physiology, it is not surprising that dysfunction of H_2_S contributes to the pathogenesis of kidney related diseases. Thereafter, administration of H_2_S mainly with NaHS was proven to rescue kidney damages in animal models with various types of kidney diseases. In the future, drug like H_2_S donors need to be tested to translate H_2_S as a treatment for renal diseases in clinical. Besides, in-depth studies of H_2_S mediated molecular actions are also needed to complete our understanding of the role of H_2_S in both renal physiology and pathology.

## Author Contributions

All authors listed, have made substantial, direct and intellectual contribution to the work, and approved it for publication.

## Conflict of Interest Statement

The authors declare that the research was conducted in the absence of any commercial or financial relationships that could be construed as a potential conflict of interest.

## Abbreviations

AC: adenylate cyclase
AMPK: 5′ AMP-activated protein kinase
AOAA: aminooxyacetic acid
CaMTg: pancreatic β-cell specific calmodulin-overexpressing transgenic
CAT: cysteine aminotransferase
CBS: cystathionine β-synthase
CKD: chronic kidney disease
CO: carbon monoxide
CSE: cystathionine γ-lyase
DAO: D-amino acid oxidase
DN: diabetic nephropathy
EDHF: endothelium-derived hyperpolarizing factor
EDRF: endothelium-derived relaxing factor
GFR: glomerular filtration rate
H_2_S: hydrogen sulfide
IRI: ischemia/reperfusion injury
JG: juxtaglomerular
MAPK: mitogen-activated protein kinase
MMP-9: matrix metalloproteinase-9
NF-κB: nuclear factor-κB
NKA: Na^+^/K^+^ ATPase
NKCC: Na^+^/K^+^/2Cl^-^ cotransporter
NO: nitric oxide
PAG: DL-propargylglycine
RAS: renin–angiotensin system
ROS: reactive oxygen species
STZ: streptozotocin
Uk⋅V: urinary potassium
UNa⋅V: urinary sodium
VEGF: vascular endothelial growth factor
3-MP: 3-mercaptopyruvate
3-MST: 3-mercaptopyruvate sulfurtransferase.

## References

[B1] Abe K. Kimura H. (1996). The possible role of hydrogen sulfide as an endogenous neuromodulator. *J. Neurosci.* 16 1066–1071. 8558235 10.1523/JNEUROSCI.16-03-01066.1996 PMC6578817

[B2] Ahangarpour A. Abdollahzade Fard A. Gharibnaseri M. K. Jalali T. Rashidi I. (2014). Hydrogen sulfide ameliorates the kidney dysfunction and damage in cisplatin-induced nephrotoxicity in rat. *Vet. Res. Forum* 5 121–127. 25568705 PMC4279637

[B3] Ahmad A. Olah G. Szczesny B. Wood M. E. Whiteman M. Szabo C. (2016). AP39, A mitochondrially targeted hydrogen sulfide donor, exerts protective effects in renal epithelial cells subjected to oxidative stress in vitro and in acute renal injury in vivo. *Shock* 45 88–97. 10.1097/SHK.0000000000000478 26513708 PMC4684477

[B4] Ahmad F. U. Sattar M. A. Rathore H. A. Abdullah M. H. Tan S. Abdullah N. A. (2012). Exogenous hydrogen sulfide (H2S) reduces blood pressure and prevents the progression of diabetic nephropathy in spontaneously hypertensive rats. *Ren. Fail.* 34 203–210. 10.3109/0886022X.2011.643365 22229751

[B5] Ahmad F. U. Sattar M. A. Rathore H. A. Tan Y. C. Akhtar S. Jin O. H. (2014). Hydrogen sulphide and tempol treatments improve the blood pressure and renal excretory responses in spontaneously hypertensive rats. *Ren. Fail.* 36 598–605. 10.3109/0886022X.2014.882218 24502512

[B6] Al-Magableh M. R. Kemp-Harper B. K. Hart J. L. (2015). Hydrogen sulfide treatment reduces blood pressure and oxidative stress in angiotensin II-induced hypertensive mice. *Hypertens. Res.* 38 13–20. 10.1038/hr.2014.125 25099489

[B7] Aminzadeh M. A. Vaziri N. D. (2012). Downregulation of the renal and hepatic hydrogen sulfide (H2S)-producing enzymes and capacity in chronic kidney disease. *Nephrol. Dial. Transplant.* 27 498–504. 10.1093/ndt/gfr560 22036943

[B8] Andresdottir G. Bakker S. J. Hansen H. P. Parving H. H. Rossing P. (2013). Urinary sulphate excretion and progression of diabetic nephropathy in Type 1 diabetes. *Diabet. Med.* 30 563–566. 10.1111/dme.12131 23324103

[B9] Azizi F. Seifi B. Kadkhodaee M. Ahghari P. (2015). Administration of hydrogen sulfide protects ischemia reperfusion-induced acute kidney injury by reducing the oxidative stress. *Ir. J. Med. Sci.* 185 649–654. 10.1007/s11845-015-1328-z 26141462

[B10] Bellomo R. Kellum J. A. Ronco C. (2012). Acute kidney injury. *Lancet* 380 756–766. 10.1016/S0140-6736(11)61454-2 22617274

[B11] Beltowski J. (2010). Hypoxia in the renal medulla: implications for hydrogen sulfide signaling. *J.* *Pharmacol. Exp. Ther.* 334 358–363. 10.1124/jpet.110.166637 20427475

[B12] Bidani A. K. Griffin K. A. (2004). Pathophysiology of hypertensive renal damage: implications for therapy. *Hypertension* 44 595–601. 10.1161/01.HYP.0000145180.38707.84 15452024

[B13] Boor P. Ostendorf T. Floege J. (2010). Renal fibrosis: novel insights into mechanisms and therapeutic targets. *Nat. Rev. Nephrol.* 6 643–656. 10.1038/nrneph.2010.120 20838416

[B14] Bos E. M. Leuvenink H. G. Snijder P. M. Kloosterhuis N. J. Hillebrands J. L. Leemans J. C. (2009). Hydrogen sulfide-induced hypometabolism prevents renal ischemia/reperfusion injury. *J. Am. Soc. Nephrol.* 20 1901–1905. 10.1681/ASN.2008121269 19628669 PMC2736772

[B15] Bos E. M. Wang R. Snijder P. M. Boersema M. Damman J. Fu M. (2013). Cystathionine gamma-lyase protects against renal ischemia/reperfusion by modulating oxidative stress. *J. Am. Soc. Nephrol.* 24 759–770. 10.1681/ASN.2012030268 23449534 PMC3636788

[B16] Bruce King S. (2013). Potential biological chemistry of hydrogen sulfide (H2S) with the nitrogen oxides. *Free Radic. Biol. Med.* 55 1–7. 10.1016/j.freeradbiomed.2012.11.005 23165065 PMC3798156

[B17] Cooper M. E. (1998). Pathogenesis, prevention, and treatment of diabetic nephropathy. *Lancet* 352 213–219. 10.1016/S0140-6736(05)79820-2 9683226

[B18] Della Coletta Francescato H. Cunha F. Q. Costa R. S. Barbosa Junior F. Boim M. A. Arnoni C. P. (2011). Inhibition of hydrogen sulphide formation reduces cisplatin-induced renal damage. *Nephrol. Dial. Transplant.* 26 479–488. 10.1093/ndt/gfq447 20656754

[B19] Dombkowski R. A. Naylor M. G. Shoemaker E. Smith M. DeLeon E. R. Stoy G. F. (2011). Hydrogen sulfide (H(2)S) and hypoxia inhibit salmonid gastrointestinal motility: evidence for H(2)S as an oxygen sensor. *J. Exp. Biol.* 214(Pt 23) 4030–4040. 10.1242/jeb.061473 22071195

[B20] Dronavalli S. Duka I. Bakris G. L. (2008). The pathogenesis of diabetic nephropathy. *Nat. Clin. Pract. Endocrinol. Metab.* 4 444–452. 10.1038/ncpendmet0894 18607402

[B21] Dursun M. Otunctemur A. Ozbek E. Sahin S. Besiroglu H. Ozsoy O. D. (2015). Protective effect of hydrogen sulfide on renal injury in the experimental unilateral ureteral obstruction. *Int. Braz. J. Urol.* 41 1185–1193. 10.1590/S1677-5538.IBJU.2014.0090 26742979 PMC4756947

[B22] Edwards G. Feletou M. Weston A. H. (2012). Hydrogen sulfide as an endothelium-derived hyperpolarizing factor in rodent mesenteric arteries. *Circ. Res.* 110 e13–e14. 22223214 10.1161/CIRCRESAHA.111.259309

[B23] Eltzschig H. K. Eckle T. (2011). Ischemia and reperfusion [mdash] from mechanism to translation. *Nat. Med.* 17 1391–1401. 22064429 10.1038/nm.2507 PMC3886192

[B24] Feliers D. Lee H. J. Kasinath B. S. (2016). Hydrogen sulfide in renal physiology and disease. *Antioxid. Redox Signal.* 10.1089/ars.2015.6596 [Epub ahead of print]. PMC5079410 27005700

[B25] Fu M. Zhang W. Wu L. Yang G. Li H. Wang R. (2012). Hydrogen sulfide (H2S) metabolism in mitochondria and its regulatory role in energy production. *Proc. Natl. Acad. Sci. U.S.A.* 109 2943–2948. 10.1073/pnas.1115634109 22323590 PMC3287003

[B26] Gambaryan S. Wagner C. Smolenski A. Walter U. Poller W. Haase W. (1998). Endogenous or overexpressed cGMP-dependent protein kinases inhibit cAMP-dependent renin release from rat isolated perfused kidney, microdissected glomeruli, and isolated juxtaglomerular cells. *Proc. Natl. Acad. Sci. U.S.A.* 95 9003–9008. 10.1073/pnas.95.15.9003 9671794 PMC21192

[B27] Ge S. N. Zhao M. M. Wu D. D. Chen Y. Wang Y. Zhu J. H. (2014). Hydrogen sulfide targets EGFR Cys797/Cys798 residues to induce Na(+)/K(+)-ATPase endocytosis and inhibition in renal tubular epithelial cells and increase sodium excretion in chronic salt-loaded rats. *Antioxid. Redox Signal.* 21 2061–2082. 10.1089/ars.2013.5304 24684506 PMC4215382

[B28] Gorin Y. Cavaglieri R. C. Khazim K. Lee D. Y. Bruno F. Thakur S. (2015). Targeting NADPH oxidase with a novel dual Nox1/Nox4 inhibitor attenuates renal pathology in type 1 diabetes. *Am. J. Physiol. Renal Physiol.* 308 F1276–F1287. 10.1152/ajprenal.00396.2014 25656366 PMC4451325

[B29] Guidotti T. L. (2010). Hydrogen sulfide: advances in understanding human toxicity. *Int. J. Toxicol.* 29 569–581. 10.1177/1091581810384882 21076123

[B30] Han S. J. Kim J. I. Park J. W. Park K. M. (2015). Hydrogen sulfide accelerates the recovery of kidney tubules after renal ischemia/reperfusion injury. *Nephrol. Dial. Transplant.* 30 1497–1506. 10.1093/ndt/gfv226 26142397

[B31] Hart P. D. Bakris G. L. (2010). Hypertensive nephropathy: prevention and treatment recommendations. *Expert Opin. Pharmacother.* 11 2675–2686. 10.1517/14656566.2010.485612 20718588

[B32] Hirose Y. (2010). [Clinical aspects of hydrogen sulfide poisoning]. *Chudoku Kenkyu* 23 212–216. 20865906

[B33] Holwerda K. M. Burke S. D. Faas M. M. Zsengeller Z. Stillman I. E. Kang P. M. (2014). Hydrogen sulfide attenuates sFlt1-induced hypertension and renal damage by upregulating vascular endothelial growth factor. *J. Am. Soc. Nephrol.* 25 717–725. 10.1681/ASN.2013030291 24335973 PMC3968492

[B34] House J. D. Brosnan M. E. Brosnan J. T. (1997). Characterization of homocysteine metabolism in the rat kidney. *Biochem. J.* >328(Pt 1) 287–292. 9359866 10.1042/bj3280287 PMC1218919

[B35] Hu H. Shi Y. Chen Q. Yang W. Zhou H. Chen L. (2008). Endogenous hydrogen sulfide is involved in regulation of respiration in medullary slice of neonatal rats. *Neuroscience* 156 1074–1082. 10.1016/j.neuroscience.2008.08.025 18793700

[B36] Hunter J. P. Hosgood S. A. Patel M. Rose R. Read K. Nicholson M. L. (2012). Effects of hydrogen sulphide in an experimental model of renal ischaemia-reperfusion injury. *Br. J. Surg.* 99 1665–1671. 10.1002/bjs.8956 23132416

[B37] Ishii I. Akahoshi N. Yu X. N. Kobayashi Y. Namekata K. Komaki G. (2004). Murine cystathionine gamma-lyase: complete cDNA and genomic sequences, promoter activity, tissue distribution and developmental expression. *Biochem. J.* 381(Pt 1) 113–123. 15038791 10.1042/BJ20040243 PMC1133768

[B38] Jiang D. Zhang Y. Yang M. Wang S. Jiang Z. Li Z. (2014). Exogenous hydrogen sulfide prevents kidney damage following unilateral ureteral obstruction. *Neurourol. Urodyn.* 33 538–543. 10.1002/nau.22450 23784934

[B39] Kamat P. K. Kalani A. Tyagi S. C. Tyagi N. (2015). Hydrogen sulfide epigenetically attenuates homocysteine-induced mitochondrial toxicity mediated through NMDA receptor in mouse brain endothelial (bEnd3) cells. *J. Cell. Physiol.* 230 378–394. 10.1002/jcp.24722 25056869 PMC4305357

[B40] Kaur M. Sachdeva S. Bedi O. Kaur T. Kumar P. (2015). Combined effect of hydrogen sulphide donor and losartan in experimental diabetic nephropathy in rats. *J. Diabetes Metab. Disord.* 14:63 10.1186/s40200-015-0212-8 PMC4517497 26221579

[B41] Kimura H. (2011). Hydrogen sulfide: its production, release and functions. *Amino Acids* 41 113–121. 10.1007/s00726-010-0510-x 20191298

[B42] Kimura H. (2014). The physiological role of hydrogen sulfide and beyond. *Nitric Oxide* 41 4–10. 10.1016/j.niox.2014.01.002 24491257

[B43] Koning A. M. Frenay A. R. Leuvenink H. G. van Goor H. (2015). Hydrogen sulfide in renal physiology, disease and transplantation–the smell of renal protection. *Nitric Oxide* 46 37–49. 10.1016/j.niox.2015.01.005 25656225

[B44] Kundu S. Pushpakumar S. B. Tyagi A. Coley D. Sen U. (2013). Hydrogen sulfide deficiency and diabetic renal remodeling: role of matrix metalloproteinase-9. *Am. J. Physiol. Endocrinol. Metab.* 304 E1365–E1378. 10.1152/ajpendo.00604.2012 23632630 PMC3680700

[B45] Lee H. J. Mariappan M. M. Feliers D. Cavaglieri R. C. Sataranatarajan K. Abboud H. E. (2012). Hydrogen sulfide inhibits high glucose-induced matrix protein synthesis by activating AMP-activated protein kinase in renal epithelial cells. *J. Biol. Chem.* 287 4451–4461. 10.1074/jbc.M111.278325 22158625 PMC3281646

[B46] Lee S. W. Cheng Y. Moore P. K. Bian J. S. (2007). Hydrogen sulphide regulates intracellular pH in vascular smooth muscle cells. *Biochem. Biophys. Res. Commun.* 358 1142–1147. 10.1016/j.bbrc.2007.04.203 17531202

[B47] Lee Z. W. Zhou J. Chen C. S. Zhao Y. Tan C. H. Li L. (2011). The slow-releasing hydrogen sulfide donor, GYY4137, exhibits novel anti-cancer effects in vitro and in vivo. *PLoS ONE* 6:e21077 10.1371/journal.pone.0021077 PMC3119065 21701688

[B48] Levey A. S. Coresh J. (2012). Chronic kidney disease. *Lancet* 379 165–180. 10.1016/S0140-6736(11)60178-5 21840587

[B49] Li H. Feng S. J. Zhang G. Z. Wang S. X. (2014). Correlation of lower concentrations of hydrogen sulfide with atherosclerosis in chronic hemodialysis patients with diabetic nephropathy. *Blood Purif.* 38 188–194. 10.1159/000368883 25531647

[B50] Li L. Hsu A. Moore P. K. (2009). Actions and interactions of nitric oxide, carbon monoxide and hydrogen sulphide in the cardiovascular system and in inflammation–a tale of three gases! *Pharmacol. Ther.* 123 386–400. 10.1016/j.pharmthera.2009.05.005 19486912

[B51] Li L. Whiteman M. Guan Y. Y. Neo K. L. Cheng Y. Lee S. W. (2008). Characterization of a novel, water-soluble hydrogen sulfide-releasing molecule (GYY4137): new insights into the biology of hydrogen sulfide. *Circulation* 117 2351–2360. 10.1161/CIRCULATIONAHA.107.753467 18443240

[B52] Li N. Chen L. Muh R. W. Li P. L. (2006). Hyperhomocysteinemia associated with decreased renal transsulfuration activity in Dahl S rats. *Hypertension* 47 1094–1100. 10.1161/01.HYP.0000217972.80731.ef 16636197

[B53] Lim J. J. Liu Y. H. Khin E. S. Bian J. S. (2008). Vasoconstrictive effect of hydrogen sulfide involves downregulation of cAMP in vascular smooth muscle cells. *Am. J. Physiol. Cell Physiol.* 295 C1261–C1270. 10.1152/ajpcell.00195.2008 18787076

[B54] Lin S. Visram F. Liu W. Haig A. Jiang J. Mok A. (2016). GYY4137, a slow-releasing hydrogen sulfide donor, ameliorates renal damage associated with chronic obstructive uropathy. *J. Urol.* 10.1016/j.juro.2016.05.029 [Epub ahead of print]. 27177428

[B55] Liu M. Jia Z. Sun Y. Zhang A. Yang T. (2016). A H 2 S donor GYY4137 exacerbates cisplatin-induced nephrotoxicity in mice. *Mediators Inflamm.* 2016:8145785 10.1155/2016/8145785 PMC4906217 27340345

[B56] Liu Y. Zhao H. Qiang Y. Qian G. Lu S. Chen J. (2015). Effects of hydrogen sulfide on high glucose-induced glomerular podocyte injury in mice. *Int. J. Clin. Exp. Pathol.* 8 6814–6820. 26261567 PMC4525901

[B57] Liu Y. H. Bian J. S. (2010). Bicarbonate-dependent effect of hydrogen sulfide on vascular contractility in rat aortic rings. *Am. J. Physiol. Cell Physiol.* 299 C866–C872. 10.1152/ajpcell.00105.2010 20660164

[B58] Liu Y. H. Lu M. Hu L. F. Wong P. T. Webb G. D. Bian J. S. (2012). Hydrogen sulfide in the mammalian cardiovascular system. *Antioxid. Redox. Signal.* 17 141–185. 10.1089/ars.2011.4005 22304473

[B59] Łowicka E. Bełtowski J. (2006). Hydrogen sulfide (H2S)-the third gas of interest for pharmacologists. *Pharmacol. Rep.* 59 4–24. 17377202

[B60] Lu M. Liu Y. H. Goh H. S. Wang J. J. Yong Q. C. Wang R. (2010). Hydrogen sulfide inhibits plasma renin activity. *J. Am. Soc. Nephrol.* 21 993–1002. 10.1681/ASN.2009090949 20360313 PMC2900962

[B61] Lu M. Liu Y. H. Ho C. Y. Tiong C. X. Bian J. S. (2012). Hydrogen sulfide regulates cAMP homeostasis and renin degranulation in As4.1 and rat renin-rich kidney cells. *Am. J. Physiol. Cell Physiol.* 302 C59–C66. 10.1152/ajpcell.00341.2010 21940660

[B62] Mathai J. C. Missner A. Kugler P. Saparov S. M. Zeidel M. L. Lee J. K. (2009). No facilitator required for membrane transport of hydrogen sulfide. *Proc. Natl. Acad. Sci. U.S.A.* 106 16633–16638. 10.1073/pnas.0902952106 19805349 PMC2757810

[B63] Meng G. Wang J. Xiao Y. Bai W. Xie L. Shan L. (2015a). GYY4137 protects against myocardial ischemia and reperfusion injury by attenuating oxidative stress and apoptosis in rats. *J. Biomed. Res.* 29 203–213. 10.7555/JBR.28.20140037 26060444 PMC4449488

[B64] Meng G. Zhu J. Xiao Y. Huang Z. Zhang Y. Tang X. (2015b). Hydrogen sulfide donor GYY4137 protects against myocardial fibrosis. *Oxid. Med. Cell. Longev.* 2015:691070 10.1155/2015/691070 PMC4442292 26078813

[B65] Modis K. Coletta C. Erdelyi K. Papapetropoulos A. Szabo C. (2013). Intramitochondrial hydrogen sulfide production by 3-mercaptopyruvate sulfurtransferase maintains mitochondrial electron flow and supports cellular bioenergetics. *FASEB J.* 27 601–611. 10.1096/fj.12-216507 23104984

[B66] Mustafa A. K. Sikka G. Gazi S. K. Steppan J. Jung S. M. Bhunia A. K. (2011). Hydrogen sulfide as endothelium-derived hyperpolarizing factor sulfhydrates potassium channels. *Circ. Res.* 109 1259–1268. 10.1161/CIRCRESAHA.111.240242 21980127 PMC3234531

[B67] Nagpure B. V. Bian J. S. (2016). Interaction of Hydrogen Sulfide with Nitric Oxide in the Cardiovascular System. *Oxid. Med. Cell. Longev.* 2016:6904327 10.1155/2016/6904327 PMC4657111 26640616

[B68] Nicholson R. A. Roth S. H. Zhang A. Zheng J. Brookes J. Skrajny B. (1998). Inhibition of respiratory and bioenergetic mechanisms by hydrogen sulfide in mammalian brain. *J. Toxicol. Environ. Health A* 54 491–507. 10.1080/009841098158773 9661914

[B69] Oh G. S. Pae H. O. Lee B. S. Kim B. N. Kim J. M. Kim H. R. (2006). Hydrogen sulfide inhibits nitric oxide production and nuclear factor-kappaB via heme oxygenase-1 expression in RAW264.7 macrophages stimulated with lipopolysaccharide. *Free Radic. Biol. Med.* 41 106–119. 10.1016/j.freeradbiomed.2006.03.021 16781459

[B70] Olson K. R. (2015). Hydrogen sulfide as an oxygen sensor. *Antioxid. Redox Signal.* 22 377–397. 10.1089/ars.2014.5930 24801248 PMC4307036

[B71] Olson K. R. Dombkowski R. A. Russell M. J. Doellman M. M. Head S. K. Whitfield N. L. (2006). Hydrogen sulfide as an oxygen sensor/transducer in vertebrate hypoxic vasoconstriction and hypoxic vasodilation. *J. Exp. Biol.* 209(Pt 20) 4011–4023. 10.1242/jeb.02480 17023595

[B72] Olson K. R. Whitfield N. L. (2010). Hydrogen sulfide and oxygen sensing in the cardiovascular system. *Antioxid. Redox Signal.* 12 1219–1234. 10.1089/ars.2009.2921 19803742

[B73] Pabla N. Dong Z. (2008). Cisplatin nephrotoxicity: mechanisms and renoprotective strategies. *Kidney Int.* 73 994–1007. 10.1038/sj.ki.5002786 18272962

[B74] Pan W. J. Fan W. J. Zhang C. Han D. Qu S. L. Jiang Z. S. (2015). H2S, a novel therapeutic target in renal-associated diseases? *Clin. Chim. Acta* 438 112–118. 10.1016/j.cca.2014.08.005 25149103

[B75] Peres L. A. B. da Cunha A. D. Jr. (2013). Acute nephrotoxicity of cisplatin: molecular mechanisms. *J. Bras. Nefrol.* 35 332–340. 10.5935/0101-2800.20130052 24402113

[B76] Peters J. Münter K. Bader M. Hackenthal E. Mullins J. Ganten D. (1993). Increased adrenal renin in transgenic hypertensive rats, TGR (mREN2) 27, and its regulation by cAMP, angiotensin II, and calcium. *J. Clin. Invest.* 91 742–747. 10.1172/JCI116292 8383701 PMC288023

[B77] Qian X. Li X. Ma F. Luo S. Ge R. Zhu Y. (2016). Novel hydrogen sulfide-releasing compound, S-propargyl-cysteine, prevents STZ-induced diabetic nephropathy. *Biochem. Biophys. Res. Commun.* 473 931–938. 27055593 10.1016/j.bbrc.2016.03.154

[B78] Reiffenstein R. J. Hulbert W. C. Roth S. H. (1992). Toxicology of hydrogen sulfide. *Annu. Rev. Pharmacol. Toxicol.* 32 109–134. 10.1146/annurev.pa.32.040192.000545 1605565

[B79] Roy A. Khan A. H. Islam M. T. Prieto M. C. Majid D. S. (2012). Interdependency of cystathione gamma-lyase and cystathione beta-synthase in hydrogen sulfide-induced blood pressure regulation in rats. *Am. J. Hypertens.* 25 74–81. 10.1038/ajh.2011.149 21866187 PMC3258007

[B80] Safar M. M. Abdelsalam R. M. (2015). H2S donors attenuate diabetic nephropathy in rats: modulation of oxidant status and polyol pathway. *Pharmacol. Rep.* 67 17–23. 10.1016/j.pharep.2014.08.001 25560570

[B81] Schweda F. Friis U. Wagner C. Skott O. Kurtz A. (2007). Renin release. *Physiology* 22 310–319. 10.1152/physiol.00024.2007 17928544

[B82] Shibuya N. Koike S. Tanaka M. Ishigami-Yuasa M. Kimura Y. Ogasawara Y. (2013). A novel pathway for the production of hydrogen sulfide from D-cysteine in mammalian cells. *Nat. Commun.* 4:1366 10.1038/ncomms2371 23340406

[B83] Shibuya N. Tanaka M. Yoshida M. Ogasawara Y. Togawa T. Ishii K. (2009). 3-Mercaptopyruvate sulfurtransferase produces hydrogen sulfide and bound sulfane sulfur in the brain. *Antioxid. Redox Signal.* 11 703–714. 10.1089/ARS.2008.2253 18855522

[B84] Simon F. Scheuerle A. Groger M. Stahl B. Wachter U. Vogt J. (2011). Effects of intravenous sulfide during porcine aortic occlusion-induced kidney ischemia/reperfusion injury. *Shock* 35 156–163. 10.1097/SHK.0b013e3181f0dc91 20661185

[B85] Smith R. P. Gosselin R. E. (1979). Hydrogen sulfide poisoning. *J. Occup. Med.* 21 93–97. 10.1097/00043764-197902000-00008 556262

[B86] Song K. Wang F. Li Q. Shi Y. B. Zheng H. F. Peng H. (2014). Hydrogen sulfide inhibits the renal fibrosis of obstructive nephropathy. *Kidney Int.* 85 1318–1329. 10.1038/ki.2013.449 24284510 PMC4040941

[B87] Stipanuk M. H. (2004). Sulfur amino acid metabolism: pathways for production and removal of homocysteine and cysteine. *Annu. Rev. Nutr.* 24 539–577. 10.1146/annurev.nutr.24.012003.132418 15189131

[B88] Stipanuk M. H. Beck P. W. (1982). Characterization of the enzymic capacity for cysteine desulphhydration in liver and kidney of the rat. *Biochem. J.* 206 267–277. 7150244 10.1042/bj2060267 PMC1158582

[B89] Stipanuk M. H. King K. M. (1982). Characteristics of the enzymatic capacity for cysteine desulfhydration in cat tissues. *Comp. Biochem. Physiol. B* 73 595–601. 10.1016/0300-9629(82)90266-3 6960976

[B90] Szabo C. (2007). Hydrogen sulphide and its therapeutic potential. *Nat. Rev. Drug Discov.* 6 917–935. 10.1038/nrd2222 17948022

[B91] Tang G. Yang G. Jiang B. Ju Y. Wu L. Wang R. (2013). H(2)S is an endothelium-derived hyperpolarizing factor. *Antioxid. Redox Signal.* 19 1634–1646. 10.1089/ars.2012.4805 23418650

[B92] Teng H. Wu B. Zhao K. Yang G. Wu L. Wang R. (2013). Oxygen-sensitive mitochondrial accumulation of cystathionine beta-synthase mediated by Lon protease. *Proc. Natl. Acad. Sci. U.S.A.* 110 12679–12684. 10.1073/pnas.1308487110 23858469 PMC3732959

[B93] Tripatara P. Patel N. S. Brancaleone V. Renshaw D. Rocha J. Sepodes B. (2009). Characterisation of cystathionine gamma-lyase/hydrogen sulphide pathway in ischaemia/reperfusion injury of the mouse kidney: an in vivo study. *Eur. J. Pharmacol.* 606 205–209. 10.1016/j.ejphar.2009.01.041 19374832

[B94] Tripatara P. Patel N. S. Collino M. Gallicchio M. Kieswich J. Castiglia S. (2008). Generation of endogenous hydrogen sulfide by cystathionine gamma-lyase limits renal ischemia/reperfusion injury and dysfunction. *Lab. Invest.* 88 1038–1048. 10.1038/labinvest.2008.73 18679378

[B95] van den Born J. C. Frenay A. R. Bakker S. J. Pasch A. Hillebrands J. L. Lambers Heerspink H. J. (2016). High urinary sulfate concentration is associated with reduced risk of renal disease progression in type 2 diabetes. *Nitric Oxide* 55–56, 18–24. 10.1016/j.niox.2016.03.001 26952289

[B96] Wallace J. L. Wang R. (2015). Hydrogen sulfide-based therapeutics: exploiting a unique but ubiquitous gasotransmitter. *Nat. Rev. Drug Discov.* 14 329–345. 10.1038/nrd4433 25849904

[B97] Wang Q. Song B. Jiang S. Liang C. Chen X. Shi J. (2015). Hydrogen sulfide prevents advanced glycation end-products induced activation of the epithelial sodium channel. *Oxid. Med. Cell. Longev.* 2015:976848 10.1155/2015/976848 PMC4442307 26078825

[B98] Wang R. (2002). Two’s company, three’s a crowd: can H2S be the third endogenous gaseous transmitter? *FASEB J.* 16 1792–1798. 10.1096/fj.02-0211hyp 12409322

[B99] Wang R. (2009). Hydrogen sulfide: a new EDRF. *Kidney Int.* 76 700–704. 10.1038/ki.2009.221 19536086

[B100] Warenycia M. W. Smith K. A. Blashko C. S. Kombian S. B. Reiffenstein R. J. (1989). Monoamine oxidase inhibition as a sequel of hydrogen sulfide intoxication: increases in brain catecholamine and 5-hydroxytryptamine levels. *Arch. Toxicol.* 63 131–136. 2730337 10.1007/BF00316435

[B101] Xia M. Chen L. Muh R. W. Li P. L. Li N. (2009). Production and actions of hydrogen sulfide, a novel gaseous bioactive substance, in the kidneys. *J. Pharmacol. Exp. Ther.* 329 1056–1062. 10.1124/jpet.108.149963 19246614 PMC2683781

[B102] Xu Z. Prathapasinghe G. Wu N. Hwang S. Y. Siow Y. L. Oh K. (2009). Ischemia-reperfusion reduces cystathionine-beta-synthase-mediated hydrogen sulfide generation in the kidney. *Am. J. Physiol. Renal Physiol.* 297 F27–F35. 10.1152/ajprenal.00096.2009 19439522

[B103] Xue H. Yuan P. Ni J. Li C. Shao D. Liu J. (2013). H(2)S inhibits hyperglycemia-induced intrarenal renin-angiotensin system activation via attenuation of reactive oxygen species generation. *PLoS ONE* 8:e74366 10.1371/journal.pone.0074366 PMC3772925 24058553

[B104] Yamamoto J. Sato W. Kosugi T. Yamamoto T. Kimura T. Taniguchi S. (2013). Distribution of hydrogen sulfide (H(2)S)-producing enzymes and the roles of the H(2)S donor sodium hydrosulfide in diabetic nephropathy. *Clin. Exp. Nephrol.* 17 32–40. 10.1007/s10157-012-0670-y 22872231 PMC3572382

[B105] Yang G. Wu L. Jiang B. Yang W. Qi J. Cao K. (2008). H2S as a physiologic vasorelaxant: hypertension in mice with deletion of cystathionine gamma-lyase. *Science* 322 587–590. 10.1126/science.1162667 18948540 PMC2749494

[B106] Yong Q. C. Pan T. T. Hu L. F. Bian J. S. (2008). Negative regulation of beta-adrenergic function by hydrogen sulphide in the rat hearts. *J. Mol. Cell Cardiol.* 44 701–710. 10.1016/j.yjmcc.2008.01.007 18329040

[B107] Yu F. Zhao J. Tang C. S. Geng B. (2010). [Effect of synthesized GYY4137, a slowly releasing hydrogen sulfide donor, on cell viability and distribution of hydrogen sulfide in mice]. *Beijing Da Xue. Xue. Bao* 42 493–497. 20957002

[B108] Yuan P. Xue H. Zhou L. Qu L. Li C. Wang Z. (2011). Rescue of mesangial cells from high glucose-induced over-proliferation and extracellular matrix secretion by hydrogen sulfide. *Nephrol. Dial. Transplant.* 26 2119–2126. 10.1093/ndt/gfq749 21208996

[B109] Yuzawa Y. (2012). [Role of hydrogen sulfide in chronic kidney disease and diabetic nephropathy]. *Nihon Yakurigaku Zasshi* 139 17–21. 10.1254/fpj.139.17 22230876

[B110] Zhang J. Chen S. Liu H. Zhang B. Zhao Y. Ma K. (2013). Hydrogen sulfide prevents hydrogen peroxide-induced activation of epithelial sodium channel through a PTEN/PI(3,4,5)P3 dependent pathway. *PLoS ONE* 8:e64304 10.1371/journal.pone.0064304 PMC3669336 23741314

[B111] Zhang X. Bian J. S. (2014). Hydrogen sulfide: a neuromodulator and neuroprotectant in the central nervous system. *ACS Chem. Neurosci.* 5 876–883. 10.1021/cn500185g 25230373

[B112] Zhou X. Feng Y. Zhan Z. Chen J. (2014). Hydrogen sulfide alleviates diabetic nephropathy in a streptozotocin-induced diabetic rat model. *J. Biol. Chem.* 289 28827–28834. 10.1074/jbc.M114.596593 25164822 PMC4200243

[B113] Zhu J. X. Kalbfleisch M. Yang Y. X. Bihari R. Lobb I. Davison M. (2012). Detrimental effects of prolonged warm renal ischaemia-reperfusion injury are abrogated by supplemental hydrogen sulphide: an analysis using real-time intravital microscopy and polymerase chain reaction. *BJU Int.* 110(11 Pt C) E1218–E1227. 10.1111/j.1464-410X.2012.11555.x 23046222

